# Microbial mechanisms of tolerance to weak acid stress

**DOI:** 10.3389/fmicb.2013.00416

**Published:** 2013-12-30

**Authors:** Nuno P. Mira, Miguel C. Teixeira

**Affiliations:** Biological Sciences Research Group, Department of Bioengineering, Institute for Biotechnology and Bioengineering, Instituto Superior Técnico, Universidade de LisboaLisbon, Portugal

**Keywords:** carboxylic acids, weak acid food preservatives, response and resistance to weak acid stress, food-borne spoilage microbes, tolerance to weak acids

Carboxylic acids are ubiquitous molecules found in microbial metabolic pathways and that have been explored for a wide array of applications including food preservation (e.g., acetic, propionic, benzoic, and sorbic acids), chemotherapy (e.g., the analgesic acetylsalicylic acid, the immunosuppressor mycophenolic acid or the antimalarial drugs artesunic and artemisinic acids) or agriculture (e.g., the herbicides 2,4-dichlorophenoxyacetic acid). This research topic contributes to the understanding of the molecular mechanisms underlying adaptation to weak acid stress in microbes [extensively reviewed in Piper et al. (2001); Boot et al. (2002); Cotter and Hill (2003); Smits and Brul (2005); Mollapour et al. (2008); Teixeira et al. (2011)], a knowledge base that impacts the fields of Medicine, Health, Food Safety, and the Environment.

The exploitation of carboxylic acids as “building-block molecules” for chemical synthesis has recently been a hot topic of research, accompanying the orientation of US and EU policy towards the development of biorefineries (Sauer et al., 2008; Abbott et al., 2009). To be economically sustainable biomass-based refineries must efficiently produce biofuels, but must also produce effective alternatives to the oil-derivatives that are used today as precursors/catalysts by the chemical industry. The carboxylic group, along with other functional groups frequently found in carboxylic acids, make these molecules attractive platforms for chemical synthesis and/or catalysis. The efforts put by the scientific community to develop efficient processes for large-scale production of carboxylic acids from renewable carbon sources, exploring different microbes as cell factories, have led to significant improvements (Sauer et al., 2008; Abbott et al., 2009); however, the yield of microbial carboxylic acid production is still limited. This low yield is mainly caused by to the toxic effects of the acids in the producing cells and by the diversion of substrate towards metabolites other than the acid of interest. The article of Jarboe et al. (2013) reviews the toxic effects exerted by, and the underlying adaptive responses to, lipophilic carboxylic acids in *Saccharomyces cerevisiae* and *Escherichia coli*, two host systems that have been exploited as cell factories. Within the same context, Steiger et al. (2013) review the most recent findings regarding biosynthesis of itaconic acid in *Aspergillus terreus* as well as the metabolic- and genetic engineering-based strategies attempted to improve the yield of production of this acid in this fungus and in other hosts.

The ability of microbial pathogens to colonize the human body is frequently dependent on their ability to tolerate carboxylic acids. This the case of probiotic bacteria that need to tolerate the presence of bile salts (composed by bile acids conjugated with glycine or taurine) to colonize the gut, or of *Candida* species that need to cope with acetic and lactic acids in the vaginal tract, produced by the bacteria that co-colonize that niche. Ruiz et al. (2013) review the main adaptive responses of probiotic Lactobacilli and Bifidobacteria to cope with the toxic effect exerted by bile salts and by bile. Several reports on adaptive mechanisms to weak acids in *Candida* are featured as well. The involvement of the multidrug (MDR) transporter of the Major Facilitator Superfamily (MFS) CgAqr1 in *C. glabrata* tolerance to acetic acid and to the antifungal drugs flucytosine and clotrimazole is herein demonstrated (Costa et al., 2013). The involvement of the *HOG1*-signalling pathway, controlled by the Hog1 protein kinase, in *C. glabrata* tolerance and response to sorbic acid is described by Jandric et al. (2013). Sorbic acid is widely used as a preservative in over-the-counter vaginal products and therefore the identification of genes/proteins involved in *C. glabrata* resistance to this compound is detrimental to improve the efficacy of those products and, consequently, to control infections caused by this pathogenic yeast.

Weak carboxylic acids (e.g., acetic, propionic, sorbic and benzoic acids) are important food preservatives and spoilage microbes must overcome their presence to grow in food products. Much of what is known today regarding the mechanisms underlying tolerance and resistance to weak acid food preservatives in spoilage Fungi, particularly at a genome-wide scale, was gathered in the experimental eukaryotic model yeast *Saccharomyces cerevisiae*, itself a spoilage yeast (Piper et al., 2001; Mollapour et al., 2008; Mira et al., 2010). Two studies dedicated to *S. cerevisae* responses to weak acid stress are included in this research topic. The review of Giannattasio et al. (2013) focuses on the equilibrium between the activation of pro-survival or pro-death mechanisms described to occur in acetic acid-stressed yeast cells. The second study, undertaken by Ullah et al. (2013), shows that under extreme weak acid stress *S. cerevisiae* cells prefer to preserve energy reserves thus limiting the activation of energy-consuming adaptive mechanisms (such as the activity of the plasma membrane proton pump). Results from Ullah et al. also reinforce a previous hypothesis that yeast cells adapt to weak acid stress by modifying the cell envelope to reduce the diffusion rate of the undissociated acid to the intracellular environment (Simoes et al., 2006; Mira et al., 2009). The study of Diakogiannis et al. (2013) shows that this mechanism of diffusional restriction is also relevant for protection against weak acid toxicity in the food-borne pathogen *Listeria monocytogenes*. The response of *Campylobacter jejuni*, also a food-borne pathogen, to formic acid is the scope of the study performed by Kassem et al. (2013). Formic acid has been used as a feed additive to reduce emergence of *C. jejuni* in chickens, the primary reservoir of this pathogen. However, the data obtained suggest that formic acid might act as an inducer (or at least a positive modulator) of entry of *C. jejuni* cells into a viable-but-not-culturable (VBNC) state that allows these bacterial cells to become untraceable. The development of a pH-sensitive fluorescent probe to monitor the internal pH of *Bacillus subtillis* cells is the goal of the study of Van Beilen and Brul (2013). Using this newly developed probe the authors demonstrate that germination of *B. subtillis* spores involves a prominent increase in internal pH up to 7 (Van Beilen and Brul, 2013). In the presence of inhibitory concentrations was not altered of sorbic acid in the extracellular milieu reduced the germination rate and abrogated the increase in internal pH, while no significant effect was registered under acetic acid stress (Van Beilen and Brul, 2013). It thus seems that sorbic acid is more suited to prevent contamination of food products with *B. subtillis* than acetic acid.

Altogether this research topic highlights the importance of pursuing the in-depth study of the molecular mechanisms underlying the toxicity and resistance to weak organic acids as an important way to contribute for the development of more appropriate tools for the control and elimination of food-borne pathogens/contaminants that thrive in acidic environments and for the engineering of optimized strains to be used as superior cell factories, able to tolerate inhibitory weak acid concentrations, among other fermentation-related stresses.
